# Thymectomy lowers the myasthenia gravis biomarker miR-150-5p

**DOI:** 10.1212/NXI.0000000000000450

**Published:** 2018-03-01

**Authors:** Carl Johan Molin, Liis Sabre, Cleo-Aron Weis, Tanel Punga, Anna Rostedt Punga

**Affiliations:** From the Department of Neuroscience, Clinical Neurophysiology (C.J.M., L.S., A.R.P.), Uppsala University, Sweden; Institute of Pathology (C.-A.W.), University Medical Centre Mannheim, Medical Faculty Mannheim, University of Heidelberg, Germany; and Department of Medical Biochemistry and Microbiology (T.P.), Uppsala University, Sweden.

## Abstract

**Objective:**

The aim of the study was to analyze the effect of thymectomy on the proposed disease-specific microRNA (miRNA) biomarkers miR-150-5p and miR-21-5p in patients from the prospective randomized trial of thymectomy in myasthenia gravis (MGTX trial) and to evaluate the longitudinal changes in clinical patterns compared with these miRNA levels.

**Methods:**

Serum samples were obtained from 80 patients with MG who were included in the MGTX trial. Thirty-eight patients were randomized to thymectomy plus prednisone treatment, and 42 patients were randomized to prednisone treatment. Serum samples were analyzed for the expression of miR-150-5p and miR-21-5p, with quantitative reverse transcriptase PCR at baseline and at 12, 24, and 36 months after randomization. The inclusion criteria for participation in the MGTX trial were age 18–65 years, generalized myasthenia gravis (Myasthenia Gravis Foundation of America Class II–IV), disease duration of less than 5 years, and seropositivity for acetylcholine receptor antibodies (AChR+).

**Results:**

Patients treated with thymectomy had lower levels of miR-150-5p at 24 months, both compared with baseline values (*p* = 0.0011) and the prednisone group (*p* = 0.04). No change in miRNA levels was found in the prednisone group. Levels of miR-21-5p displayed a negative correlation with the prednisone dose within the prednisone-only group (*p* ≤ 0.001).

**Conclusions:**

Thymectomy lowers the levels of the proposed biomarker miR-150-5p, which strengthens its position as a potential disease-specific biomarker for AChR+ MG.

Myasthenia gravis (MG) is a chronic autoimmune neuromuscular disorder, in which patients have autoantibodies against the nicotinic acetylcholine receptor (AChR+, 85%), muscle-specific tyrosine kinase, or low-density lipoprotein receptor–related protein 4.^1,2^ The thymus plays a central role in the pathogenesis of MG, and thymic abnormalities are most frequently found among AChR+ patients.^3^ The randomized trial of thymectomy in myasthenia gravis^4^ (MGTX trial) supports thymectomy for AChR+ patients with generalized MG.

MicroRNAs (miRNAs) are small, noncoding RNA molecules that regulate gene expression in mammalian cells. These miRNAs can be exported from the cells and are detected as extracellular circulating entities in various biofluids.^5,6^ Because of their simple detection and alterations in diseases, circulating miRNAs can serve as biomarkers, offering advances for earlier diagnosis, prognosis, and therapeutic monitoring of many diseases.^7,8^ As MG is largely mediated by T and B cells, miRNAs that are essential for T- and B-cell differentiation and immune response could be potentially dysregulated in patients with MG. Previous studies have shown that circulating miR-150-5p and miR-21-5p are elevated specifically in sera of AChR+ MG patients' without immunosuppression and display a disease-specific accumulation pattern.^9–11^ Considering the heterogeneous nature of the disease, as well as the fluctuation in muscle fatigue, circulating prognostic biomarkers would be valuable. The aim of the present study was to validate the biomarkers miR-150-5p and miR-21-5p through longitudinal analysis of the effect of thymectomy on these miRNAs in relation to clinical status. For this purpose, we analyzed serum samples collected from a large MG patient cohort from the prospective international MGTX trial.

## Methods

### Study design and hypothesis

The primary objective consisted of testing the hypothesis that mean miR-150-5p and miR-21-5p values would be reduced over time after thymectomy in parallel with clinical improvement. The secondary exploratory objective was to investigate the correlation between the prednisone dose and miR-150-5p or miR-21-5p within the prednisone group.

### Participants

The inclusion criteria for participating in the MGTX trial were as follows: age ranging from 18 to 65 years, generalized myasthenia gravis (Myasthenia Gravis Foundation of America Class II–IV), disease duration <5 years, and AChR antibody seropositivity (AChR+). Patients were randomized either to thymectomy plus prednisone or prednisone treatment, and we applied the same subgrouping as in the original MGTX trial. Full details on trial design and study protocol are available in the original study.^4^ In total, 126 patients were accepted into the MGTX trial. We obtained serum samples from each of the 80 patients (42 in the prednisone-only group and 38 in the thymectomy group) drawn at baseline and at 12, 24, and 36 months postrandomization. Serum samples were stored at −80°C until further processing. Clinical and demographic data, which were obtained from the MGTX study group, included age at enrollment, disease duration, prednisone nativity at enrollment, actual and time-weighted quantitative MG (QMG) score, and prednisone dose, as well as data on other treatments (plasmapheresis, intravenous immunoglobulin, and immunosuppressive medications) at each time point.

### miRNA isolation and expression analysis

A small RNA fraction was isolated from 200 μL of serum samples using the miRCURY RNA Isolation Kit-Biofluids (#300112; Exiqon, Denmark) according to the manufacturer's instructions. Two microliters of isolated RNA was used for complementary DNA (cDNA) synthesis in a 10-μL reaction mix, using the Universal cDNA Synthesis Kit II (Exiqon #203301).

Quantitative PCR (qPCR) reactions were performed with ExiLENT SYBR Green master mix (Exiqon, #203421), supplied with ROX Reference Dye (#12223-012; Life Technologies, Carlsbad, CA). The cDNA templates were diluted 50X in nuclease-free water. The qPCR reactions were applied to custom-ordered 384-well Pick-&-Mix plate (Exiqon, #203819), including validated detection primers sets for the following miRNAs: hsa-miR-150-5p, hsa-miR-21-5p, hsa-miR-103a-3p, hsa-miR-191-5p, hsa-miR-93-5p, and hsa-423-5p. In addition, detectors for hemolysis (hsa-miR-23a-3p and hsa-miR-451a), interplate calibration (UniSp3), RNA extraction (UniSp2 and UniSp4), and cDNA synthesis (UniSp6) were included on the Pick-&-Mix PCR panel plates. To simplify reading, the prefix “hsa,” which specifies human (*Homo sapiens*) miRNAs, has been omitted in the body of the text. Target amplification was performed with the Applied Biosystems 7900HT Fast Real-Time PCR System (Life Technologies). The reactions where reverse transcriptase was removed in the cDNA synthesis step were used as reaction specificity controls. To rule out serum contamination with intracellular miRNAs due to hemolysis, a ΔC_T(hemolysis)_ value was calculated (ΔC_T(hemolysis)_ = C_T(miR-23a-3p)_−C_T(miR-451a)_). A ΔC_T(hemolysis)_ value >7 indicates a high risk of hemolysis, and samples with a value higher than 7 were excluded from further analysis. The qPCR raw data were analyzed with GenEx software, supplied by Exiqon. Reference genes included in the panel were miR-93-5p, miR-191-5p, miR-423-5p, and miR-103a because they all have stable expression in the sera of patients with MG.^10^ As the reference miRNA, miR-191-5p was chosen because it was recommended by the “NormFinder” function in GenEx software. Quantification of relative miRNA expression was performed with the comparative C_T_ method using the formula 2^−ΔΔCT^, where ΔΔC_T_ = (C_T (miRNA of interest)_ − C_T (reference miRNA)_) sample A − (C_T (miRNA of interest)_ − C_T (reference miRNA)_) sample B.^12^

### Thymic lymphofollicular hyperplasia grading

Histological thymus data from the patients who had undergone thymectomy were obtained from the Department of Pathology, Medical Faculty Mannheim, and University of Heidelberg, Mannheim, Germany. Grading of thymic lymphofollicular hyperplasia (TFH) was performed with immunohistochemistry for CD23 or with hematoxylin and eosin staining when CD23 grading was missing. Follicle content was assessed as the percentage of follicle-positive area per 20 low-power fields (LPFs, ×50 magnification). In grade I, 1%–33%, in grade II 34%–66%, and in grade III 67%–100% of the LPFs are follicle positive. Furthermore, grade IV TFH is equal to more than 3 follicles per any LPF. Grade I can be observed in normal thymic tissue, whereas grades II–IV are seemed as characteristic of early-onset myasthenia gravis (EOMG). Full details on the analysis of thymus samples and classification have been published previously.^13^

### Statistical analysis

The miRNA raw data were multiplied by 100 and then log_2_-converted to obtain more normally distributed data. The QMG score and prednisone data were considered nonparametric. An unpaired 2-tailed *t* test was performed to examine between-group differences in miR-150-5p and miR-21-5p at baseline as well as at 12, 24, and 36 months postbaseline, with a null hypothesis of equal mean values. A paired, 2-tailed *t* test was performed to examine whether there was a difference in miR-150-5p and miR-21-5p levels between baseline and at 12, 24, and 36 months postbaseline within the same group, with a null hypothesis of no difference in the mean values. Only when significant, a Wilcoxon's signed rank test would also be performed to see whether the reduced miRNA level was accompanied by a reduction in the QMG score or prednisone dose to reduce the risk of error inflation. Also, a post hoc analysis of covariance (ANCOVA) was performed to test group differences in miRNA levels adjusted for baseline values, sex, and age. Spearman's rank correlation coefficient was used to determine the correlation between the miRNA, QMG score, and prednisone dose as a post hoc analysis of the subgroup of prednisone-naive patients in the prednisone group. Pearson's χ^2^ test was used to compare clinical characteristics between the groups. Post hoc subgroup analyses with *t* tests of miRNA levels were performed as follows: (1) male vs female and (2) age (<50 and ≥50 years) at enrollment. A *p* value of <0.05 was considered statistically significant. No imputation methods were used for missing data. Crossover patients (patients randomized to prednisone who underwent thymectomy and vice versa) were analyzed according to the treatment they received. All statistical analyses were performed with R version 3.2.4.^14^

### Standard protocol approvals, registrations, and patient consents

Approval for analysis of serum samples from the MGTX study was received from Uppsala ethical standards committee on human experimentation (Dnr 2010/446/2, accepted 2016-02-22). Written informed consent was obtained from all patients participated in the MGTX study.^4^

## Results

### Participants and samples

Eighty patients from the MGTX trial were included in this study. Clinical characteristics are presented in table 1. Patient serum samples were missing at 15 of 320 time points. One sample in the thymectomy group had a ΔC_T(hemolysis)_ > 7 and was thus excluded from further analysis. Furthermore, 34 samples were excluded because of miRNA levels below the detection limit (C_T_ > 38) according to the guidelines on miRNA data analysis provided in GenEx software. At baseline, no patients in either of the 2 groups received any immunosuppressive medication other than prednisone. In the thymectomy plus prednisone group, 2 patients received other immunosuppressive medications at 12 months, 5 patients at 24 months, and 7 patients at 36 months. In the prednisone group, the corresponding number of patients was 8, 17, and 20, respectively (table 2). Over the whole study period, the use of immunosuppressants other than prednisone was more prevalent in the prednisone group (N = 23) compared with the thymectomy plus prednisone group (N = 7; *p* < 0.001). The same relationship was seen after 24 months, when the use of immunosuppressants was more prevalent in the prednisone group (N = 14) compared with the thymectomy plus prednisone group (N = 2; *p* < 0.01).

**Table 1:**
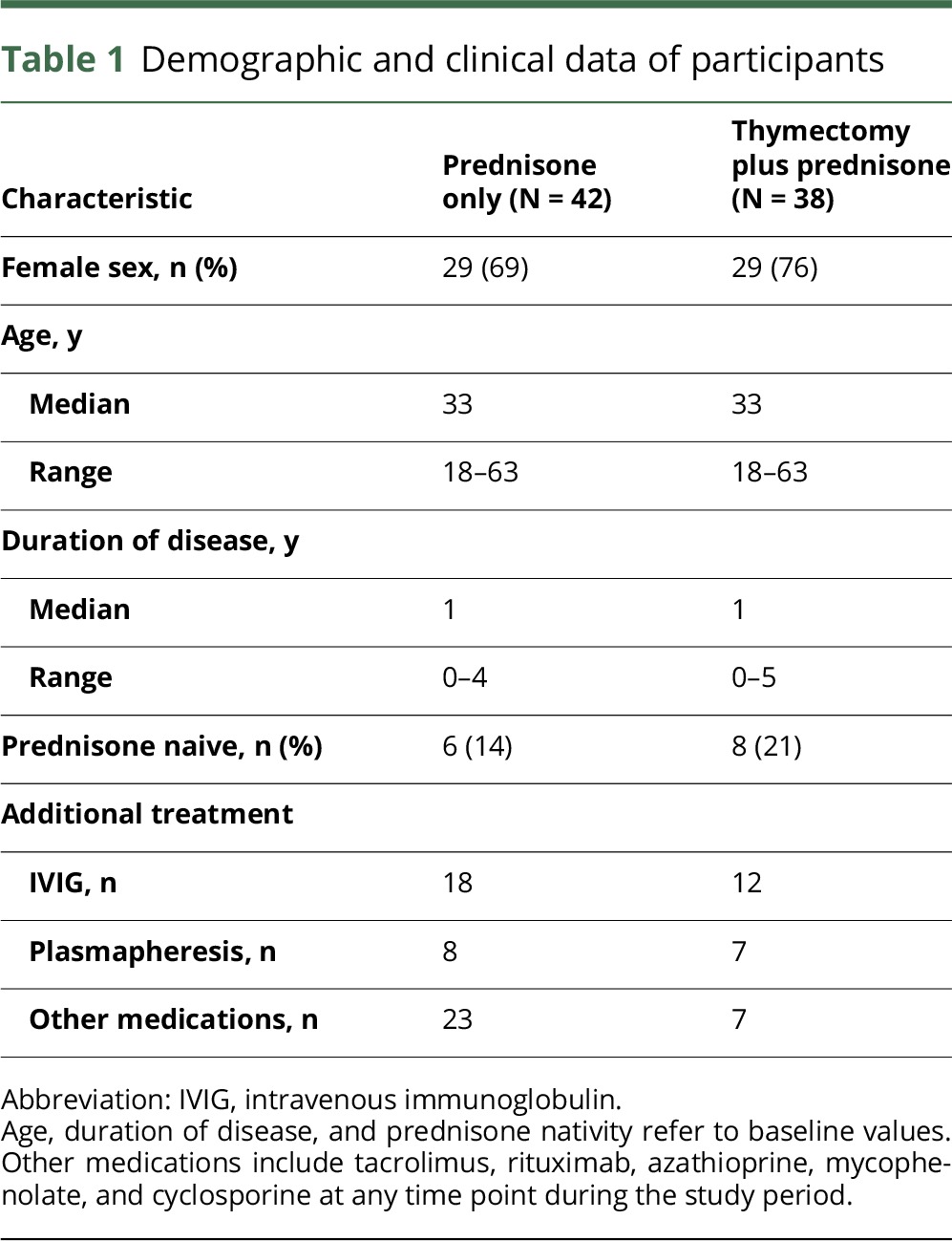
Demographic and clinical data of participants

**Table 2:**
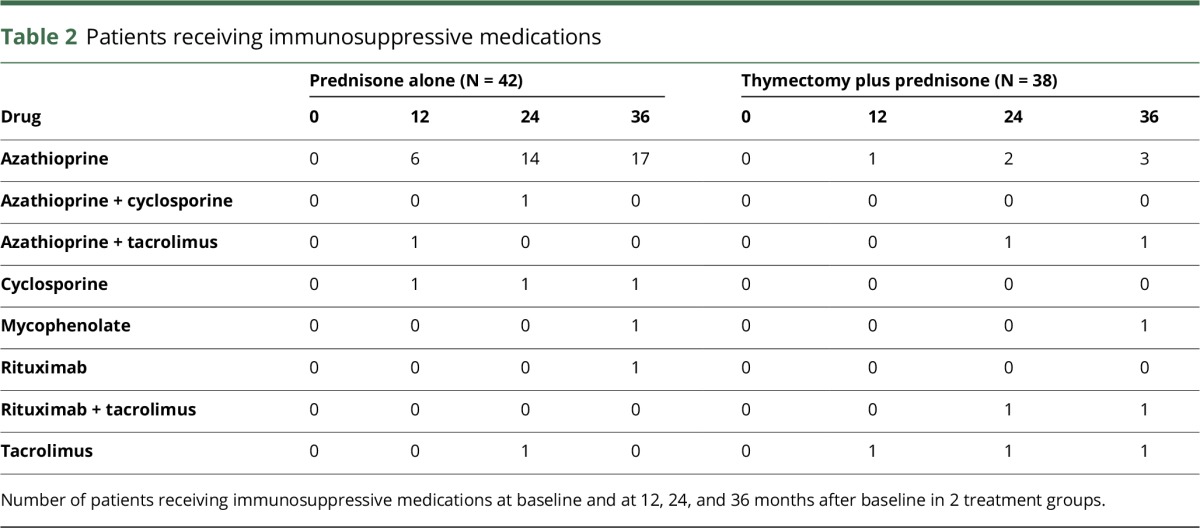
Patients receiving immunosuppressive medications

### Longitudinal analysis of circulating miR-150-5p and miR-21-5p expression in the thymectomy group

**Figure 1:**
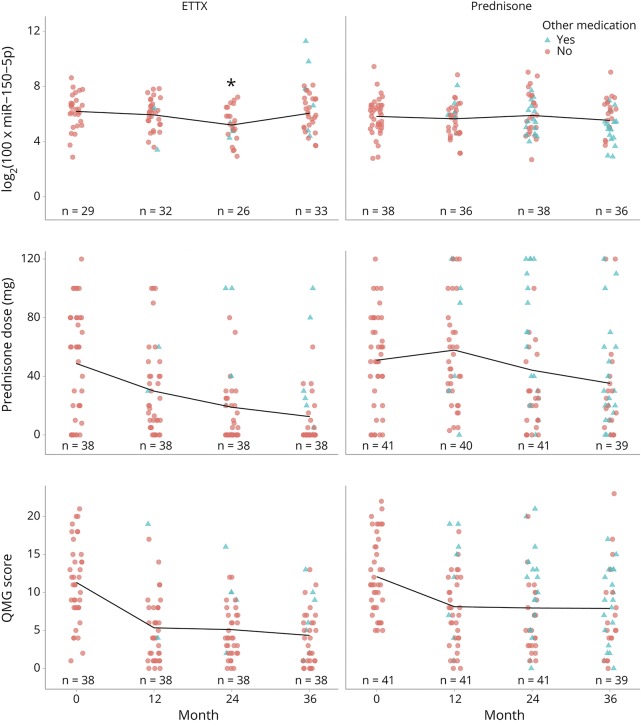
Comparison of miR-150-5p (log scale), prednisone dose (mg), and QMG score between the thymectomy (ETTX) and prednisone groups Lines represent mean values. Turquoise triangle indicates other immunosuppressant medications; the red circle indicates no other medications other than prednisone. *p ≤ 0.001. ETTX = extended transsternal thymectomy; QMG = quantitative MG.

Compared with baseline (6.2 ± 1.3), levels of miR-150-5p were clearly reduced at 24 months after thymectomy (5.2 ± 1.2; figure 1; *p* = 0.0011). The reduction in miR-150-5p levels was accompanied by the improved clinical QMG score (*p* < 0.001) and reduced prednisone dose (*p* < 0.001). Levels of miR-150-5p were reduced in 21 patients after 24 months, whereas 5 patients did not have reduced levels, 10 patients had undetectable levels (bad serum samples), and 4 samples were missing. After 36 months, miR-150-5p levels were reduced in 11 patients compared with baseline, whereas 14 patients did not have reduced levels, 5 patients had undetectable levels, and 1 sample was missing. Subgroup analysis revealed a reduction in miR-150-5p at 24 months also in patients aged <50 years (*p* = 0.0058) as well as in female patients (*p* = 0.01). Levels of miR-21-5p were not lower at 24 months but instead had a pattern of higher levels at 36 months after thymectomy in the entire cohort (*p* = 0.005), as well as in female patients (*p* = 0.02) and patients aged <50 years (*p* = 0.0082).

### Comparison of thymectomy and prednisone groups regarding miRNA expression

The longitudinal trend and relationship between miR-150-5p, prednisone dose, and QMG score in both groups are shown in figure 1. The longitudinal changes that were observed in the thymectomy group were not observed within the prednisone group. There were no differences in miR-150-5p levels between the thymectomy and the prednisone group at baseline (6.2 ± 1.3 vs 5.8 ± 1.3; *p* > 0.05) or at 12 months after randomization (5.9 ± 1.2 vs 5.7 ± 1.2; *p* > 0.05). However, patients in the thymectomy group had lower levels of miR-150-5p at 24 months after randomization (5.2 ± 1.2 vs 5.5 ± 1.3; *p* = 0.04). The same result was seen when adjusting for miRNA baseline values, sex, and age in the ANCOVA (*p* = 0.015). Nevertheless, at the later time point at 36 months, thymectomy patients had relatively higher levels of both miR-150-5p (6.3 ± 1.2 vs 5.5 ± 1.3; *p* = 0.036) and miR-21-5p (10.8 ± 1.1 vs 10.0 ± 1.3; *p* = 0.007) than prednisone-only treated patients at 36 months after randomization. The same pattern was seen in patients aged <50 years (n = 67), with lower levels in the thymectomy group of miR-150-5p at 24 months after randomization (*p* = 0.03) and higher levels of miR-21-5p at 36 months after randomization (*p* = 0.01). Levels of miR-21-5p displayed a negative correlation with the prednisone dose in prednisone-naive patients in the prednisone group (R = −0.34; *p* < 0.001). No absolute correlation was found between miRNA levels and QMG in any of the groups.

### Grade of TFH and circulating miRNA accumulation

Data on TFH were available for 35 of the 38 patients in the thymectomy group. Thirty-one percent of the patients showed thymic alterations characteristic of EOMG.

**Figure 2:**
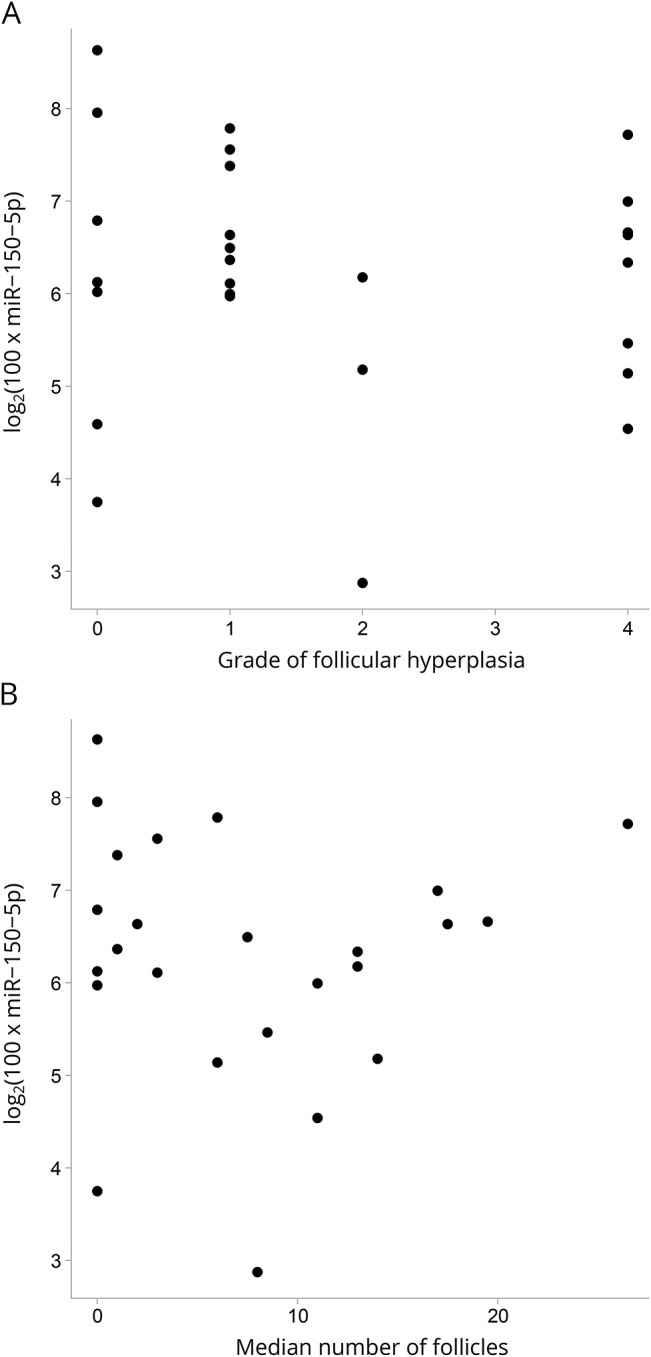
Thymus hyperplasia and miR-150-5p levels Scatter plot indicating miR-150-5p related to (A) the grade of thymic lymphofollicular hyperplasia (TFH) and (B) the median number of follicles.

Twenty-one patients had a TFH of grade I or less, i.e., no pathologic changes in the removed thymus tissue. Three patients had grade II, 0 patients had grade III, and 11 patients had grade IV. Among the patients who had TFH grade IV, all 7 patients whose miR-150-5p levels were detectable (3 patients had undetectable levels) had an increase in miR-150-5p between 24 months and 36 months. Five patients with TFH grade IV displayed a unique pattern of changes in miR-150-5p levels: reduction from baseline values to 24 months but then again an increase to 36 months after thymectomy. Among patients with TFH grade 4, baseline levels of miR-150-5p were 6.1 ± 1.1; at 24 months, levels were 5.2 ± 1.3; and at 36 months, levels were higher at 6.8 ± 1.7. No correlation was found between levels of miR-150-5p and TFH grade (figure 2A) or median number of follicles (figure 2B; *p* > 0.05 for both).

## Discussion

During the last decade, circulating blood-based miRNAs have gained the reputation of promising biomarkers for diagnosis, prognosis, and therapeutic options in a variety of disorders such as cancer, cardiovascular diseases, and diabetes among others.^7,15,16^ Based on our previous studies, circulating miR-150-5p and miR-21-5p were specifically elevated in AChR+ MG patients.^9–11,17^ Therefore, we focused on these 2 circulating miRNAs in this longitudinal study because previous studies were retrospective, nonrandomized, and performed on small homogenous patient cohorts. Importantly, therefore, this is the first randomized, prospective, single-blinded study to examine the longitudinal effect of thymectomy on the proposed serum-derived biomarkers miR-150-5p and miR-21-5p, along with the clinical QMG score, in a unique, heterogeneous population of patients with MG. The main finding was that miR-150-5p levels were lower 24 months after thymectomy, accompanied by a reduction in the QMG score, whereas no such pattern was seen in the prednisone group. This implies that a longitudinal reduction in miR-150-5p accompanies clinical improvement after thymectomy, especially in female patients aged <50 years, as supported by the subgroup analysis. This is in support of our previous study, in which we found a similar reduction in miR-150-5p in a small cohort of 9 female AChR+ MG patients who had undergone thymectomy.^10^ Another study^9^ did not find a correlation between miR-150-5p levels and the clinical MG composite score, although these patients were analyzed only at 1 particular time point. The current finding, that a reduction in miR-150-5p is accompanied by a reduction in disease severity (QMG score) at 24 months after thymectomy, further strengthens the disease-specific value of miR-150-5p in AChR+ MG. The fact that the reduction in miR-150-5p was first seen after 24 months, whereas the QMG score was reduced as early as 12 months, implies that the immunological changes from the thymectomy occurs over a longer time period, and perhaps takes longer than the more rapidly occurring clinical improvement.

As an immunoregulatory miRNA, miR-150-5p plays an important role in the development, maturation, proliferation, and survival of T cells.^18^ MiR-150-5p is upregulated during the maturation of T cells,^19^ a process in which the thymus gland is crucial, whereas miR-150-5p is downregulated during further differentiation of naive T cells.^20^ In MG, CD4+ CD25+_ FoxP3+_ regulatory T cells (Tregs) are defective.^21^ Tregs are critical to sustaining immunologic homeostasis, representing a distinct cell type that is committed to suppression of the autoimmune response.^22^ Tregs that are deficient not only in Dicer (the enzyme required for miRNA maturation) but also in Rab27 (a protein involved in vesicle release), show impaired ability to suppress the Th1 response. This suggests that non–cell-autonomous gene silencing, mediated by extracellular vesicle (EV)-associated miRNAs, is a potential mechanism used by Tregs to suppress T-cell–mediated disease.^23^ In EOMG, the number of impaired Tregs with downregulated FoxP3 is increased, but the full mechanism of how Tregs are contributing to the development of EOMG is not yet known.^24^ It was recently shown that the miRNA profile of Treg-derived EVs showed enrichment of miR-150-5p and miR-21-5p, thus differentiating the Treg EVs from T effector (Th1/Th17) cell–derived EVs.^25^ Female patients with generalized early-onset AChR+ MG most often benefit from thymectomy, and they often show TFH. However, intriguingly, we also found that all the patients with the highest TFH grade IV had an increase in miR-150-5p between 24 and 36 months. The reason for this remains unknown. Other than TFH, we found no common denominator for the patients whose miRNA levels increased from 24 to 36 months.

In previous studies, miR-21-5p had a weaker association with MG,^10^ and this is in line with the present finding that miR-21-5p did not decrease as much as miR-150-5p. The higher prevalence of immunomodulatory drugs other than prednisone in the prednisone group is a factor that could have influenced the results. A majority of patients who received immunosuppressants other than prednisone initiated treatment at the 24-month visit, and thus, this could possibly explain why the levels of miR-150-5p and miR-21-5p were lower in the prednisone group after 36 months. On the contrary, the use of other immunosuppressants, particularly azathioprine, was also less prevalent in the thymectomy group after 24 months compared with the prednisone group. Therefore, the fact that miR-150-5p levels were still lower in the thymectomy group at that time point strengthens the hypothesis that actually thymectomy alone reduces miR-150-5p levels. Furthermore, the negative correlation between miR-21-5p levels and the prednisone dose in non–prednisone-naive patients in the prednisone-only group indicates that circulating miRNA levels may respond differently to various immunosuppressive treatments. Considering the positive effect of thymectomy as an intervention, it is not surprising that we did not see this correlation in the whole group. This, and the fact that the thymectomy group has both a lower QMG score and a lower requirement for prednisone,^4^ indicating a lower autoimmune response, means that the non–prednisone-naive patients in the prednisone group may be considered a more homogenous group.

A reliable biomarker should be easy to measure and have a stable expression in a heterogeneous group of patients and ideally should correlate with the disease state. Such biomarkers are at the moment lacking for patients with MG.^26^ The AChR antibody titer does not correlate well with the state of the disease, neither between patients nor between different time points in the same patient. Because of the fluctuating nature of MG, the clinical state can vary quite considerably, even during the course of 1 day, and muscle fatigue is therefore not a good parameter to follow clinically to gain a long-term perspective or for use in clinical trials. The result that thymectomy lowers miR-150-5p, regardless of the clinical heterogeneity of the AChR+ study group, not only strengthens its value as a disease biomarker but also is yet another piece of evidence supporting the clinical effect of thymectomy in patients with MG.

The low number of prednisone-naive patients at randomization may be considered as a drawback of this study; however, this could not be avoided because of the MGTX study design. Because both prednisone and thymectomy have an effect on the immune response, they most certainly also have an effect on the expression of miR-150-5p. As mentioned previously,^13^ this is probably also an explanation for the low number of higher grade TFH. To truly examine the effect of thymectomy and prednisone treatment on miRNA, it would have been ideal to have all patients prednisone naive at baseline.

In conclusion, this is the first ever study to profile the circulating miR-150-5p longitudinally in a unique, randomized MG patient group. The results that both miR-150-5p and QMG score are lower 24 months after thymectomy provide new evidence and further support the use of miR-150-5p as a biomarker in MG. Nevertheless, further studies are needed to determine whether miR-150-5p is a reliable biomarker of clinical function over time in AChR+ MG.

## Glossary

AChR: acetylcholine receptor
ANCOVA: analysis of covariance
cDNA: complementary DNA
EOMG: early-onset myasthenia gravis
EV: extracellular vesicle
LPF: low-power field
miRNA: microRNA
MG: myasthenia gravis
MGTX trial: randomized trial of thymectomy in myasthenia gravis
qPCR: quantitative PCR
QMG: quantitative MG
TFH: thymic lymphofollicular hyperplasia
